# Nurse-coordinated biomarker and symptom monitoring for immune checkpoint inhibitor toxicity

**DOI:** 10.3389/fmed.2026.1949715

**Published:** 2026-09-11

**Authors:** Yun Feng, Feng Yuan, Shenlian Zhao

**Affiliations:** 1 School of Nursing, Zhejiang Chinese Medical University, Hangzhou, Zhejiang, China; 2 Department of Anesthesiology and Operating Room, Zhejiang Hospital, Hangzhou, Zhejiang, China; 3 Department of Thoracic Surgery, Zhejiang Hospital, Hangzhou, Zhejiang, China

**Keywords:** biomarkers, early warning, immune checkpoint inhibitors, immune-related adverse events, laboratory surveillance, nursing, patient safety, symptom monitoring

## Abstract

Immune checkpoint inhibitors have transformed cancer treatment, but immune-related adverse events (irAEs) may affect almost any organ and can progress rapidly when early manifestations are overlooked. Because nurses perform repeated assessment, laboratory review, symptom triage, patient education, and coordination across treatment cycles, nurse-coordinated monitoring is a clinically relevant component of ICI safety. This targeted narrative Mini Review distinguishes four biomarker functions: pretreatment prediction of future irAE risk, longitudinal surveillance, diagnostic support after symptoms or laboratory abnormalities emerge, and severity or outcome prognostication. Complete blood count-derived indices, C-reactive protein, albumin, lactate dehydrogenase, ferritin, cytokines, autoantibodies, and immune-cell subsets provide non-interchangeable signals, while organ-directed tests may identify thyroid, adrenal, hepatic, renal, pancreatic, cardiac, neuromuscular, or pulmonary injury. We discuss the immunobiological links between checkpoint blockade, loss of peripheral tolerance, autoreactive lymphocytes, cytokine amplification, and measurable laboratory changes. We further propose an implementation framework based on baseline assessment, standardized serial testing, trend interpretation, symptom correlation, electronic patient-reported outcomes, and predefined escalation pathways. Here, “early warning” means recognition of a reproducible symptom or biomarker trajectory that prompts evaluation before Common Terminology Criteria for Adverse Events grade progression or hospitalization; it does not imply universally presymptomatic detection. No single biomarker currently provides sufficient sensitivity or specificity for routine prediction or diagnosis. Biomarker trends should therefore complement, not replace, clinical assessment and responsible medical decision-making.

## 1 Introduction

Immune checkpoint inhibitors (ICIs) targeting PD-1, PD-L1, CTLA-4, and related inhibitory pathways have become integral to the treatment of multiple malignancies. Their benefits are accompanied by immune-related adverse events (irAEs), which differ from conventional cytotoxic toxicities because they arise from immune dysregulation and can involve the skin, gastrointestinal tract, liver, endocrine glands, lungs, heart, kidneys, musculoskeletal system, and nervous system. Major clinical guidelines emphasize baseline evaluation, repeated laboratory assessment, prompt grading, and multidisciplinary management (1–3). A recent large systematic review estimated mean event rates of approximately 40% for any-grade irAEs and 20% for high-grade events, defined here as Common Terminology Criteria for Adverse Events (CTCAE) grade 3–5, with higher risks during combination ICI therapy (4). Comparative evidence also indicates that individual agents have distinct serious-toxicity profiles (5).

Early recognition is difficult. Fatigue, appetite loss, cough, diarrhea, weakness, headache, and mild laboratory abnormalities may be attributed to cancer, infection, comorbidity, or concomitant treatment. irAEs may also emerge after treatment interruption and can persist for months or years (6, 7). Laboratory and immune biomarkers therefore offer a complementary source of objective information. Reviews of irAE biology and biomarkers describe potential signals from routine blood counts, inflammatory proteins, organ-function tests, cytokines, autoantibodies, and immune-cell phenotypes, but no single marker has achieved sufficient validation for universal prediction (8–11). In a retrospective single-center cohort of 418 patients, female sex, antibiotic exposure, higher post-treatment neutrophil-to-lymphocyte ratio, and higher baseline circulating tumor-cell burden were associated with irAE occurrence, whereas lower prognostic nutritional index, higher body mass index, and higher post-treatment lactate dehydrogenase were associated with greater severity (12). These observational findings remain context dependent and require external validation.

Nurses are positioned at the interface between serial laboratory data and evolving patient symptoms. They review results before treatment, identify changes between visits, conduct telephone or digital triage, reinforce self-monitoring, and escalate concerns to oncology and organ specialists. This Mini Review focuses on ICI-associated irAEs, the setting in which biomarker evidence is most developed, and examines how routine and emerging biomarkers can be incorporated into nurse-coordinated early-warning and safety-monitoring pathways.

## 2 Review methodology

This Mini Review used a targeted narrative approach rather than a systematic-review protocol. Relevant evidence was identified through targeted searches of PubMed/MEDLINE and major clinical guideline sources, including ASCO, SITC, ESMO, and ESE, with emphasis on publications available through August 2026. Search concepts included immune checkpoint inhibitors, immune-related adverse events, biomarkers, laboratory monitoring, nursing surveillance, patient-reported outcomes, and digital monitoring. We prioritized clinical guidelines, systematic reviews and meta-analyses, prospective and real-world clinical studies, and nursing or digital implementation studies directly relevant to irAE detection and monitoring. Evidence was selected through a structured, topic-focused process and synthesized qualitatively by biomarker type, organ system, intended function, and nursing application. No formal systematic screening or risk-of-bias tool was applied.

## 3 Immunobiological basis of irAEs and biomarker release

Checkpoint blockade restores antitumor T-cell activity but simultaneously reduces peripheral tolerance. Proposed mechanisms include expansion of autoreactive T-cell clones, altered regulatory T-cell function, B-cell activation, autoantibody production, cytokine amplification, molecular mimicry, and tissue cross-reactivity (6, 8). These mechanisms are not mutually exclusive and may differ by organ, ICI class, tumor type, and host susceptibility.

Several recent studies illustrate how immune dysregulation becomes measurable. High anti-PD-1 antibody occupancy on circulating CD4⁺ T cells has been associated with active irAEs (13), and a pretreatment composite incorporating effector-memory CD4⁺ T cells and T-cell receptor clonality has shown potential for risk detection (14). Autoantibody evidence is heterogeneous. A focused review summarizes the emerging literature (15), while primary cohort studies have associated baseline or pre-existing autoantibody profiles with organ-specific, earlier, or more frequent irAEs (16–18). These signals are not sufficiently specific for indiscriminate screening, but they support the principle that humoral immune activation may precede overt toxicity.

**Figure 1:**
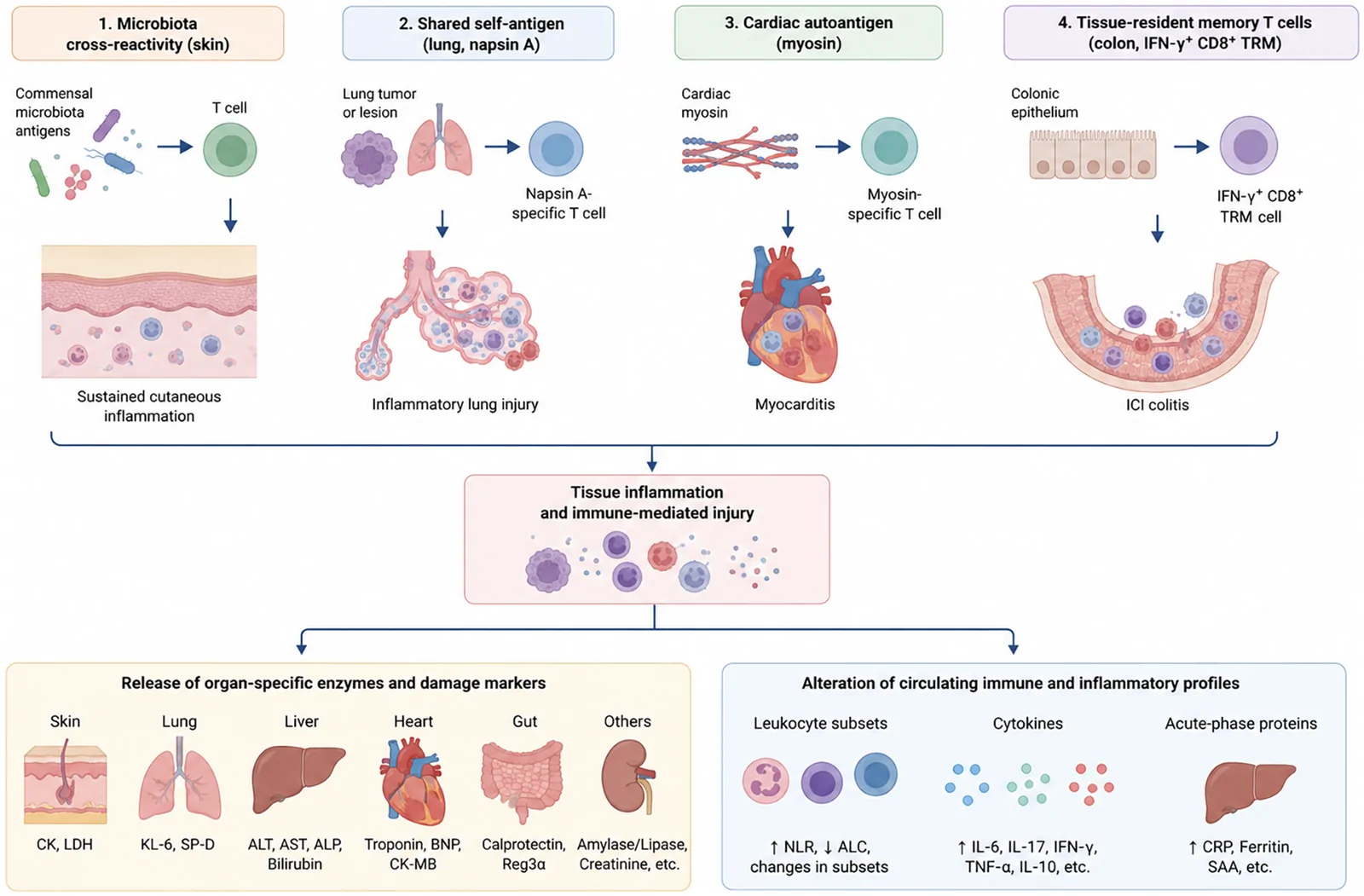
Representative mechanisms and biomarker consequences of ICI-related tissue injury. Microbiota cross-reactivity, shared self-antigens, cardiac autoantigens, and tissue-resident memory T cells may contribute to organ-specific inflammation, accompanied by tissue-damage markers and systemic changes in leukocyte subsets, cytokines, and acute-phase proteins.

Tissue injury may also arise when immune responses recognize antigens shared by tumor and normal tissue. Pathogenic responses against commensal microbiota can sustain cutaneous inflammation (19); napsin A-specific T cells have been identified in both lung tumors and inflammatory lung lesions (20); cardiac myosin-specific T cells can contribute to ICI-associated myocarditis (21); and interferon-gamma-producing CD8⁺ tissue-resident memory T cells are prominent in ICI colitis (22). The resulting tissue inflammation releases organ-specific enzymes and damage markers while altering circulating leukocyte subsets, cytokines, and acute-phase proteins. These representative mechanisms and their measurable systemic and organ-specific consequences are summarized in Figure 1.

## 4 Routine laboratory biomarkers: accessible signals with limited specificity

Routine laboratory tests are attractive because they are inexpensive, repeatable, and already embedded in treatment workflows. Complete blood count-derived indices such as neutrophil-to-lymphocyte ratio (NLR), platelet-to-lymphocyte ratio (PLR), systemic immune-inflammation index (SII), absolute lymphocyte count (ALC), and eosinophil count have been evaluated as irAE biomarkers (9–11). Their biological rationale is plausible: lymphocyte expansion may reflect immune activation, neutrophilia may reflect systemic inflammation or corticosteroid exposure, and eosinophilia may accompany selected endocrine, pulmonary, or cutaneous events. However, these indices are influenced by infection, tumor progression, marrow suppression, recent surgery, and concomitant medications.

C-reactive protein, albumin, lactate dehydrogenase, ferritin, and selected cytokines provide additional information on systemic inflammation. Rising C-reactive protein or ferritin may precede or accompany clinically important toxicity, but neither distinguishes an irAE from infection. Interleukin-6 and other cytokines are mechanistically relevant, yet assay availability, preanalytical variation, and uncertain thresholds limit routine deployment (8–10). The practical value of these markers lies in temporal concordance: a new symptom accompanied by a reproducible biomarker trajectory deserves more urgent assessment than an isolated abnormality without clinical change.

Autoantibodies and immune-cell assays may refine selected cases. Baseline antinuclear, thyroid, or disease-specific autoantibodies can identify pre-existing autoimmunity, and interval seroconversion may support an immune-mediated process (15–18). Flow-cytometric assessment of lymphocyte subsets or therapeutic-antibody occupancy is promising (13, 14), but it remains a specialized adjunct rather than a standard nursing-triggered test. Routine surveillance should therefore use a tiered model: broadly available tests for all patients, organ-directed tests when symptoms or trends emerge, and advanced immune assays in research settings or diagnostically complex cases.

## 5 Organ-directed biomarker panels

The clinical meaning of a biomarker depends on the organ at risk. Endocrine irAEs may be clinically subtle and can become permanent. European guidance recommends systematic endocrine testing, including thyroid function and context-dependent assessment of pituitary and adrenal axes (23). Meta-analytic evidence confirms increased risks of hypothyroidism, hyperthyroidism, thyroiditis, hypophysitis, diabetes, and adrenal insufficiency with PD-1 inhibition (24). In nursing practice, trends in thyroid-stimulating hormone (TSH) and free thyroxine (FT4) should be interpreted with fatigue, weight change, temperature intolerance, pulse, and bowel symptoms. Glucose, ketones, electrolytes, morning cortisol, and adrenocorticotropic hormone become urgent when polyuria, nausea, hypotension, or unexplained weakness develops. In a single-center retrospective series of 23 ICI-induced diabetes cases, cycle-by-cycle glucose monitoring was associated with a lower proportion of severe diabetic ketoacidosis (DKA); this small observational study did not establish causation and requires prospective validation (25).

For hepatic, gastrointestinal, and pancreatic toxicity, serial alanine aminotransferase, aspartate aminotransferase, alkaline phosphatase, bilirubin, albumin, amylase, and lipase should be linked to symptoms and alternative causes. A real-world cohort showed that formal causality assessment excluded non-ICI causes in a meaningful proportion of suspected checkpoint inhibitor-induced liver injury (26). A recent systematic review similarly emphasized the heterogeneous presentation of luminal, hepatobiliary, and pancreatic irAEs (27). Laboratory deterioration should therefore trigger structured evaluation rather than automatic attribution to immunotherapy.

Pulmonary toxicity illustrates the limits of blood testing. Oxygen saturation, respiratory rate, and symptom change remain central, while bronchoalveolar lavage may show CD8⁺-predominant lymphocytosis in diagnostically difficult pneumonitis (28). Krebs von den Lungen-6 (KL-6) has shown potential as a serial marker in selected non-lung-cancer populations but performs less well in patients with non-small cell lung cancer (29). These findings support conditional, not universal, use of KL-6.

Cardiac and neuromuscular toxicities require a lower threshold for escalation because early symptoms may be mild despite substantial risk. Troponin, creatine kinase (CK), CK-MB, B-type natriuretic peptide (BNP) or N-terminal pro-BNP, electrocardiography, and symptom assessment should be interpreted together. Retrospective myocarditis cohorts describe the clinical time course and prognostic importance of troponin elevation but do not validate universal surveillance thresholds (30). In selected populations, prospective high-sensitivity troponin-T monitoring has demonstrated feasibility, although assay variation and false-positive elevations limit generalizability (31). Inflammatory indices may correlate with cardiotoxicity severity (32). Creatinine, urinalysis, and electrolyte trends support renal surveillance, while new rash, pruritus, weakness, sensory change, or confusion should prompt targeted dermatologic, neurologic, or rheumatologic evaluation. Cutaneous irAEs are common and heterogeneous, reinforcing the need for direct skin assessment rather than reliance on laboratory markers alone (33).

## 6 Nurse-coordinated integration of biomarker and symptom monitoring

A nurse-coordinated monitoring pathway should begin before the first ICI dose. Baseline assessment should document autoimmune disease, endocrine and cardiopulmonary comorbidity, prior organ dysfunction, medications, infection risk, pretreatment symptoms, and reference laboratory values. The baseline laboratory set should specify complete blood count, liver-function tests, renal-function tests, glucose, electrolytes, and thyroid tests according to the ICI regimen and local protocol rather than use an undefined “biochemistry” panel. Results should be reviewed at consistent time points whenever feasible, because comparability improves interpretation of trends. Any new abnormality should be evaluated against the patient's baseline, recent treatment, symptoms, and alternative explanations (Table 1).

**Table 1:** Operational framework for nurse-coordinated biomarker and symptom monitoring during immune checkpoint inhibitor therapy.

Organ/system and biomarkers	Purpose, status, and suggested timing	Relevant symptoms and major confounders	Nurse-coordinated escalation and evidence level
General/systemic: CBC-derived NLR, PLR, SII, ALC; CRP, albumin, LDH, ferritin	Pretreatment risk prediction (exploratory), longitudinal surveillance, and severity context. Routine for CBC and standard organ-function tests; conditional for CRP/ferritin. Baseline and before cycles according to the local protocol.	Fever, fatigue, or general decline. Confounders: infection, tumor progression, marrow suppression, corticosteroids, recent surgery, and malnutrition.	Verify the trajectory and symptoms; repeat testing or request clinician review. Evidence: low to moderate, mainly reviews and retrospective cohorts (9–12).
Thyroid: TSH and FT4	Longitudinal surveillance and diagnostic support. Routine. Baseline and periodically according to guideline/local protocol.	Fatigue, weight or temperature change, pulse or bowel change. Confounders: pre-existing thyroid disease, non-thyroidal illness, and corticosteroids.	Repeat and arrange clinician/endocrine review when abnormal or symptomatic. Evidence: guideline-supported (1–3, 23, 24).
Pituitary/adrenal: morning cortisol, ACTH, sodium, potassium	Diagnostic and severity support. Conditional; symptom-triggered, with baseline testing in selected protocols.	Headache, hypotension, nausea, dizziness, or profound weakness. Confounders: exogenous corticosteroids and acute illness.	Urgent same-day medical evaluation if adrenal crisis is suspected. Evidence: guideline-supported (1–3, 23).
Glucose/diabetes: glucose, ketones, electrolytes	Surveillance and diagnostic support. Routine glucose; conditional ketones. Baseline and before cycles according to protocol; immediate testing with symptoms.	Polyuria, polydipsia, nausea, vomiting, or confusion. Confounders: pre-existing diabetes, corticosteroids, infection, and nutrition.	Same-day evaluation for marked hyperglycemia or ketosis. Evidence: guideline-supported with limited retrospective implementation data (1–3, 25).
Hepatic, gastrointestinal, pancreatic: ALT, AST, ALP, bilirubin, albumin; amylase/lipase when indicated	Surveillance, diagnostic support, and severity assessment. Routine liver tests; conditional pancreatic tests. Baseline and before cycles; symptom-triggered repeats.	Jaundice, abdominal pain, diarrhea, nausea, or fever. Confounders: infection, metastases, alcohol, concomitant drugs, biliary obstruction, and pancreatitis.	Repeat testing and clinician review; urgent assessment for rapid deterioration. Evidence: guideline/observational (1–3, 26, 27).
Pulmonary: oxygen saturation, respiratory rate; KL-6 in selected settings	Clinical surveillance and diagnostic support. Routine vital signs; KL-6 is conditional/research. At each encounter and when respiratory symptoms emerge.	Cough, dyspnea, hypoxia, or fever. Confounders: infection, lung cancer, radiotherapy, COPD, and interstitial lung disease.	Urgent clinician assessment and imaging when indicated. Evidence for KL-6 is limited and population-specific (28, 29).
Cardiac/neuromuscular: troponin, CK/CK-MB, BNP/NT-proBNP, ECG	Selected surveillance plus diagnostic/severity support. Conditional or used in high-risk/local protocols. Baseline/early-cycle testing if specified and symptom-triggered testing.	Chest pain, dyspnea, palpitations, ptosis, dysphagia, or proximal weakness. Confounders: renal dysfunction, exercise, skeletal muscle injury, tumor burden, and assay variation.	Same-day urgent clinician and cardiology/neurology evaluation. Evidence: observational with selected prospective feasibility data (30–32).
Renal, skin, neurologic/rheumatologic: creatinine, urinalysis, electrolytes plus direct clinical examination	Surveillance and diagnostic support. Routine renal tests; conditional targeted assessment. Baseline and before cycles, with symptom-triggered review.	Oliguria, edema, rash, pruritus, weakness, sensory change, confusion, or joint symptoms. Confounders: dehydration, infection, medications, and pre-existing disease.	Repeat testing and arrange organ-specific referral; emergency evaluation for rapidly progressive symptoms. Evidence: guideline-supported (1–3, 33).
Autoantibodies, cytokines, and immune-cell assays: ANA/thyroid autoantibodies; flow cytometry; selected cytokines	Pretreatment risk research, selected diagnostic support, or pharmacodynamic monitoring. Conditional/research only. Baseline or serial sampling within defined protocols.	Organ-specific symptoms. Confounders: pre-existing autoimmunity, infection, tumor-related inflammation, treatment timing, and assay variability.	Do not use as stand-alone decision tools; discuss with the clinician or relevant specialist. Evidence: exploratory and heterogeneous (13–18).

“Early warning” denotes a reproducible symptom or biomarker trajectory that prompts evaluation before CTCAE grade progression or hospitalization; it does not imply presymptomatic detection in every patient. Routine, conditional, and research status may vary by regimen, guideline, and local protocol. Biomarkers complement but do not replace clinical assessment. ACTH, adrenocorticotropic hormone; ALC, absolute lymphocyte count; ALP, alkaline phosphatase; ALT, alanine aminotransferase; ANA, antinuclear antibody; AST, aspartate aminotransferase; BNP, B-type natriuretic peptide; CBC, complete blood count; CK, creatine kinase; CRP, C-reactive protein; CTCAE, Common Terminology Criteria for Adverse Events; ECG, electrocardiogram; FT4, free thyroxine; KL-6, Krebs von den Lungen-6; LDH, lactate dehydrogenase; NLR, neutrophil-to-lymphocyte ratio; PLR, platelet-to-lymphocyte ratio; SII, systemic immune-inflammation index; TSH, thyroid-stimulating hormone.

Dynamic monitoring is more useful than a static checklist. Nurses can identify upward or downward trajectories, verify whether tests were obtained under comparable conditions, and connect laboratory signals to patient-reported changes. Operationally, “early warning” in this review means recognition of a reproducible symptom or biomarker trajectory that prompts evaluation before CTCAE grade progression or hospitalization; it does not imply presymptomatic detection in every patient. For example, a rising TSH without symptoms may justify planned reassessment, whereas the same trend with bradycardia, dizziness, or profound fatigue requires accelerated clinical review. Small troponin or CK elevations accompanied by chest discomfort, dyspnea, ptosis, or proximal weakness warrant immediate escalation. Rising liver enzymes with fever or antibiotic exposure require simultaneous evaluation for infection and drug-induced injury.

Education and accessible reporting channels are essential because many irAEs begin outside the hospital. An observational nursing study found clinically important delays between symptom onset and unscheduled hospitalization, supporting focused education and telephone triage (34). A pilot randomized study also suggested that structured education can improve management of treatment-related skin reactions (35). Teaching should use plain language, organ-specific warning symptoms, emergency contact instructions, and explicit guidance not to self-manage persistent symptoms without contacting the care team.

Electronic patient-reported outcome systems can extend surveillance between visits. A systematic review found that ICI-focused electronic symptom monitoring is feasible and acceptable, although evidence for reducing severe events remains preliminary (36). Technology-enabled monitoring has generated actionable alerts without requiring major staffing expansion in a prospective cohort (37), and randomized evaluation of nurse-led electronic monitoring models is ongoing (38). Such systems should complement, not replace, clinical judgment; overly sensitive alerts can produce alarm fatigue.

Finally, escalation pathways must be predefined. Nurses should know which biomarker or symptom changes require repeat testing, same-day clinician evaluation, emergency referral, or specialist consultation. Decisions to hold ICI therapy or initiate corticosteroids or other immunosuppression remain the responsibility of the treating medical team and should follow guideline-based grading and organ-specific assessment. Standardized multidisciplinary toxicity services have reduced recurrence and readmission for immune-mediated diarrhea and colitis (39). The nursing contribution is reliable detection, structured communication, documentation, patient advocacy, and coordination that shortens the interval from biological change to appropriate management. The proposed pathway is an evidence-informed conceptual framework that integrates guideline recommendations with observational nursing evidence, electronic patient-reported outcome studies, prospective technology-enabled monitoring, an ongoing randomized trial, and multidisciplinary service implementation (34–39). These studies provide early empirical support, but the complete end-to-end nurse-coordinated framework has not yet undergone prospective validation.

## 7 Discussion and future directions

Biomarker-informed nursing surveillance is clinically attractive, but major uncertainties remain. The evidence base is heterogeneous across study designs, tumor populations, ICI regimens, irAE definitions, assay platforms, and sampling schedules, and many candidate biomarkers derive from retrospective or single-center cohorts. Clinical implementation therefore remains an evolving field, and no biomarker panel or nurse-coordinated framework has yet achieved broad prospective validation. First, associations do not establish diagnostic specificity. Most routine markers are altered by infection, cancer progression, chemotherapy, glucocorticoids, malnutrition, or chronic disease. Trigger thresholds should therefore be used to initiate evaluation rather than to diagnose an irAE. Second, published cutoffs vary across tumor types and treatment regimens, and few models have undergone prospective external validation. Third, an irAE may coexist with antitumor benefit, but toxicity should not be treated as a desirable surrogate. Trial-level meta-analysis found weak evidence that treatment effects on irAE rates predict overall-survival effects (40), whereas patient-level studies in non-small cell lung cancer have associated mainly mild or early irAEs with favorable outcomes and severe events with worse outcomes (41). Safety decisions must remain based on toxicity severity and organ risk.

Fourth, symptom burden and laboratory change do not always evolve in parallel. Patient-reported toxicity can increase over time even when global quality of life initially improves (42). Nursing assessment should therefore retain both objective and subjective dimensions. Fifth, long-term toxicity requires follow-up beyond active treatment. Reviews show that some ICI toxicities persist or become permanent, and inconsistent reporting complicates comparisons across regimens (12, 43).

Future research should prioritize prospectively specified biomarker panels, repeated sampling, standardized preanalytical procedures, and clinically meaningful endpoints such as time to recognition, peak toxicity grade, hospitalization, treatment interruption, steroid exposure, and chronic organ dysfunction. Multivariable models should combine laboratory trajectories with symptoms, comorbidities, treatment regimen, and patient-reported outcomes rather than seek a universal single marker. Digital decision support may help visualize trends and trigger escalation, but algorithms must be interpretable, locally validated, and designed to avoid unnecessary testing and alert fatigue. Nurse-coordinated implementation studies are also needed to determine workflow feasibility, accountability, training requirements, and effects on equity for patients with limited digital access. Prospective studies should prespecify patient populations, comparator workflows, sampling schedules, assay platforms, and clinically meaningful endpoints, while testing real-world feasibility. Implementation studies should also evaluate training needs, staffing and laboratory resources, and variability across healthcare settings, together with patient-centered outcomes such as quality of life, anxiety, engagement, and monitoring burden. Successful adoption will require collaboration among nursing, oncology, laboratory medicine, and informatics. Digital systems should protect privacy, retain human oversight, and avoid widening inequities in access to advanced diagnostics or remote monitoring.

## 8 Conclusion

Routine laboratory and immune biomarkers provide a measurable link between checkpoint-induced immune dysregulation and evolving organ toxicity. Their greatest current value is not stand-alone prediction or diagnosis, but longitudinal interpretation alongside symptoms, baseline risk, treatment timing, and organ-specific context. Nurses can operationalize this information through baseline assessment, serial review, trend recognition, patient education, structured triage, and timely multidisciplinary escalation. A cautious, multimarker approach may support earlier recognition and structured evaluation, but reductions in severe toxicity, healthcare use, or treatment interruption remain to be demonstrated prospectively.
